# Detector-specific correction factors in radiosurgery beams and their impact on dose distribution calculations

**DOI:** 10.1371/journal.pone.0196393

**Published:** 2018-05-15

**Authors:** Olivia A. García-Garduño, Manuel A. Rodríguez-Ávila, José M. Lárraga-Gutiérrez

**Affiliations:** 1 Laboratorio de Física Médica, Instituto Nacional de Neurología y Neurocirugía, México City, México; 2 Posgrado en Ciencias Físicas, Instituto de Física, Universidad Nacional Autónoma de México, Ciudad Universitaria, Mexico City, México; North Shore Long Island Jewish Health System, UNITED STATES

## Abstract

Silicon-diode-based detectors are commonly used for the dosimetry of small radiotherapy beams due to their relatively small volumes and high sensitivity to ionizing radiation. Nevertheless, silicon-diode-based detectors tend to over-respond in small fields because of their high density relative to water. For that reason, detector-specific beam correction factors ($k_{Q_{clin},Q_{msr}}^{f_{clin},f_{msr}}$) have been recommended not only to correct the total scatter factors but also to correct the tissue maximum and off-axis ratios. However, the application of $k_{Q_{clin},Q_{msr}}^{f_{clin},f_{msr}}$ to in-depth and off-axis locations has not been studied. The goal of this work is to address the impact of the correction factors on the calculated dose distribution in static non-conventional photon beams (specifically, in stereotactic radiosurgery with circular collimators). To achieve this goal, the total scatter factors, tissue maximum, and off-axis ratios were measured with a stereotactic field diode for 4.0-, 10.0-, and 20.0-mm circular collimators. The irradiation was performed with a Novalis® linear accelerator using a 6-MV photon beam. The detector-specific correction factors were calculated and applied to the experimental dosimetry data for in-depth and off-axis locations. The corrected and uncorrected dosimetry data were used to commission a treatment planning system for radiosurgery planning. Various plans were calculated with simulated lesions using the uncorrected and corrected dosimetry. The resulting dose calculations were compared using the gamma index test with several criteria. The results of this work presented important conclusions for the use of detector-specific beam correction factors ($k_{Q_{clin},Q_{msr}}^{f_{clin},f_{msr}})$ in a treatment planning system. The use of $k_{Q_{clin},Q_{msr}}^{f_{clin},f_{msr}}$ for total scatter factors has an important impact on monitor unit calculation. On the contrary, the use of $k_{Q_{clin},Q_{msr}}^{f_{clin},f_{msr}}$ for tissue-maximum and off-axis ratios has not an important impact on the dose distribution calculation by the treatment planning system. This conclusion is only valid for the combination of treatment planning system, detector, and correction factors used in this work; however, this technique can be applied to other treatment planning systems, detectors, and correction factors.

## Introduction

Problems related with the dosimetry of small radiotherapy photon beams have been extensively discussed in the literature [1]. In response these problems, many detectors have been manufactured specifically for the dosimetry of small photon beams with certain advantages and disadvantages [2]. In particular, silicon-diode-based detectors have been commonly used because of their relatively small volumes and high sensitivity to ionizing radiation [3]. Moreover, silicon-diode-based detectors have been selected as the detector of choice [4–8]. However, these detectors tend to over-respond in small fields due to their high density relative to water. To minimize this over-response, the use of detector-specific beam correction factors has been proposed by Alfonso et al. [9]. By means of that approach, the correction factors can account for the difference in the detector response between small beams and the machine-specific reference field.

Detector-specific correction factors are commonly calculated with Monte Carlo simulations. The magnitude is reported for the central beam axis at a given reference depth (d) in water for a clinical field size, $f_{clin}$, relative to a machine-specific reference field size, $f_{msr}$. The correction factors ($k_{Q_{clin},Q_{msr}}^{f_{clin},f_{msr}}$) are intended to be applied to the ratio of the detector readings to determine the ratio of the absorbed dose in water between the clinical and reference fields; this ratio defines the field output factor as follows:

$$\Omega_{Q_{clin},Q_{msr}}^{f_{clin},f_{msr}}=k_{Q_{clin},Q_{msr}}^{f_{clin},f_{msr}}\frac{M_{Q_{clin}}^{f_{clin}}}{M_{Q_{msr}}^{f_{msr}}}$$ (1)

A considerable number of papers have reported $k_{Q_{clin},Q_{msr}}^{f_{clin},f_{msr}}$ values for various radiation detector and linear accelerator combinations. In particular, Francescon et al. reported the $k_{Q_{clin},Q_{msr}}^{f_{clin},f_{msr}}$ dependency of different off-axis and depths detector positions using Monte Carlo simulations for various radiation detectors used in small beam dosimetry [10]. Francescon et al. primarily defined detector correction factors for the percentage depth dose (PDD), tissue maximum ratio (TMR), and off-axis ratio (OAR) curves. These correction factors are of interest because they may modify the dosimetry data used to commission treatment planning systems (TPS) and impact the calculation of dose distributions. According to the American Association of Physicists in Medicine (AAPM) Task Group 53, an important source of uncertainty in the dose calculation is the accuracy of the original measured data [2,11]. Therefore, the impact of these factors on the calculated dose distributions performed by the TPS in the commissioning of small photon beams must be better understood.

The goal of this study is to address the impact of the detector-specific beam correction factors on the calculated dose distribution in static non-conventional photon beams (specifically, in stereotactic radiosurgery with circular collimators). The presented work was conducted in three parts. The first part consisted of measuring dosimetric data (TMR, OAR, and total scatter factors (TSF)) with a silicon stereotactic field diode (SFD) to characterize the circular collimated beams generated by a Novalis^®^ linear accelerator (LINAC). In the second part, Monte Carlo simulations were used to calculate the detector-specific beam corrections for circular collimated beams according to the formalism proposed by Alfonso et al. [9,10,12] and the dosimetry data were corrected using these factors. In the third part, the corrected and uncorrected commissioned data sets were incorporated into the TPS to compare the dose distributions calculated from the measured dosimetry. The dosimetric data obtained from the corrected and uncorrected data were compared using the gamma index, dose volume histograms, and differences of the calculated monitor units.

## Materials and methods

### A. Experimental configuration

In this study, a silicon SFD (IBA-Dosimetry, Germany) was used in the dosimetric characterization of static small photon beams. The radiation source was a Novalis^®^ LINAC (Novalis, BrainLab, Germany) with a nominal energy of 6 MV. The measurements were performed in liquid water using an MP3-XS scanning phantom (PTW-Freiburg, Germany). The measured dosimetric parameters were TMRs, OARs, and TSFs. The photon beams were collimated using conical collimators with diameters of 4.0, 10.0, and 20 mm defined at the isocenter. The TSF and OAR were experimentally determined according to the LINAC manufacturer specifications at depths of 1.5 and 7.5 cm, respectively, with a fixed source-to-axis distance of 100 cm. The TMRs were measured step-by-step at the depths of 5, 10, 15, 20, 50, 100, 150, 100, and 250 mm.

### B. Monte Carlo simulation

The Monte Carlo codes used for modeling and benchmarking the Novalis LINAC were BEAMnrc and DOSXYZnrc [13,14]. The Monte Carlo code selected for the SFD detector modeling was DOSRZnrc due to the cylindrical geometry of the problem [15,16]. This work used a previously designed general Monte Carlo model of a Novalis LINAC [12]. The original electron source parameters were as follows: 6.1-MeV monoenergetic beam with a circularly symmetric Gaussian full-width half-max (FWHM) of 1.5 mm. The collimators were modeled using the CONESTACK and SLABS modules based on detailed measurements of the geometry. The material composition of the cones was lead. Phase space files for each circular collimator were calculated such that each phase space had at least 2 million particles per square centimeter. Further details of this methodology are found in S1 File.

The SFD detector components (stainless steel stem, coaxial cable, housing, and enclosure materials) were modeled following the work of Cranmer-Sargison [15]. The SFD sensitive volume was modeled with a 0.300-mm radius on a silicon chip of radius 0.500 mm, and the geometry of the coaxial cable was approximated as a homogeneous mixture of copper and polyethylene [15].

#### B.I. Simulation of TMR, OAR, and TSF in water

**Fig 1:**
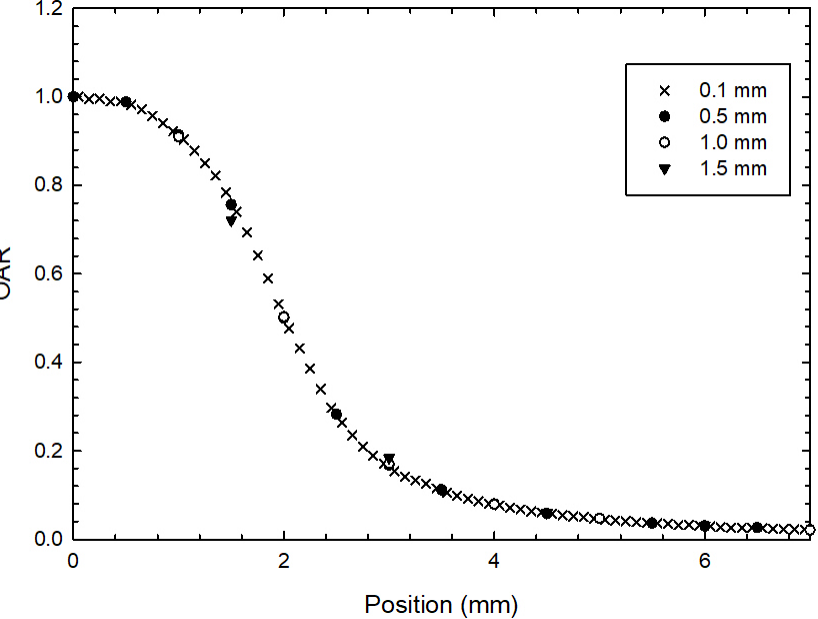
Monte Carlo calculated profiles showing the influence of voxel size on OAR shape for the 4.0-mm circular collimator. The solid line represents the 0.1-mm voxel size profile, which was chosen as a reference for the comparison.

The DOSXYZnrc code was used to calculate the absorbed dose to water in a homogenous virtual water phantom with the total dimensions of 30 cm × 30 cm × 30 cm. A voxel size of 0.5 mm × 0.5 mm × 0.5 mm was used to calculate the TMR, OAR, and TSF for each circular collimator modeled in this work. A preliminary study on the influence of voxel size on OAR was performed. Fig 1 shows the Monte Carlo calculated profiles for a 4.0-mm circular collimator with different voxel side lengths ranging from 0.1 to 1.5 mm. The profiles corresponding to 0.1 and 0.5 mm have the lowest differences (<0.2%), while the profile corresponding with the 1.5-mm voxel size clearly shows partial volume effects. According to this preliminary study, the 0.5-mm voxel size was selected because the volume-averaging effect was expected to be smaller for larger cones. The EGSnrc transport parameters used for the simulations were ECUT = 0.521 MeV, PCUT = 0.01 MeV, XCOM cross-section database, and EXACT boundary crossing.

#### B.II. Determination of detector-specific beam correction factors $k_{Q_{clin},\;Q_{msr}}^{f_{clin},\;f_{msr}}$

The central, off-axis, and in-depth correction factors were calculated using the DOSRZnrc code. Assuming that the dosimeter readings are directly proportional to the absorbed dose within the active volume of the detector, the correction factor at the central beam axis for a reference depth (d) is determined as follows:

$$k_{Q_{clin},\;Q_{msr}}^{f_{clin},\;f_{msr}}=\frac{D_{w,\;Q_{clin}}^{f_{clin}}/D_{w,Q_{msr}}^{f_{msr}}}{D_{det,Q_{clin}}^{f_{clin}}/D_{det,Q_{msr}}^{f_{msr}}}$$ (2)

where *$D_{w}$* and $D_{det}$ are the absorbed dose in water and the detector, respectively, for the clinical $f_{clin}$ and reference ${\;f}_{msr}$ fields.

Variations of the correction factors with depth in the phantom and with distance of the central axis were calculated following the methods proposed by Francescon et al. [10]. The correction factors can be calculated by

$$k_{Q_{clin},\;Q_{msr}}^{f_{clin},\;f_{msr}}\left(0,\;z,\;TMR\right)=\frac{D_{w,\;Q_{clin}}^{f_{clin}}\left(0,z\right)\cdot D_{det,Q_{msr}}^{f_{msr}}\left(0,z_{ref}\right)}{D_{det,Q_{clin}}^{f_{clin}}\left(0,z\right)\cdot D_{w,Q_{msr}}^{f_{msr}}\left(0,z_{ref}\right)}$$ (3)

$$k_{Q_{clin},\;Q_{msr}}^{f_{clin},\;f_{msr}}\left(r,\;z,\;OAR\right)=\frac{D_{w,\;Q_{clin}}^{f_{clin}}\left(r,z\right)\cdot D_{det,Q_{msr}}^{f_{msr}}\left(0,z_{ref}\right)}{D_{det,Q_{clin}}^{f_{clin}}\left(r,z\right)\cdot D_{w,Q_{msr}}^{f_{msr}}\left(0,z_{ref}\right)}$$ (4)

where D(r,z) is the dose scored within the sensitive volume of the detector in water at depth z and distance r from the beam central axis (off-axis distance).

#### B.III. Validation of SFD Monte Carlo modeling

Finally, the SFD Monte Carlo modeling was validated. The validation consisted of comparing the experimental TMR, OAR, and TSF data set to the corresponding Monte-Carlo-calculated TMR, OAR and TSF data sets with the SFD model included in the simulation.

### C. Planning simulation (dose distribution analysis)

**Fig 2:**
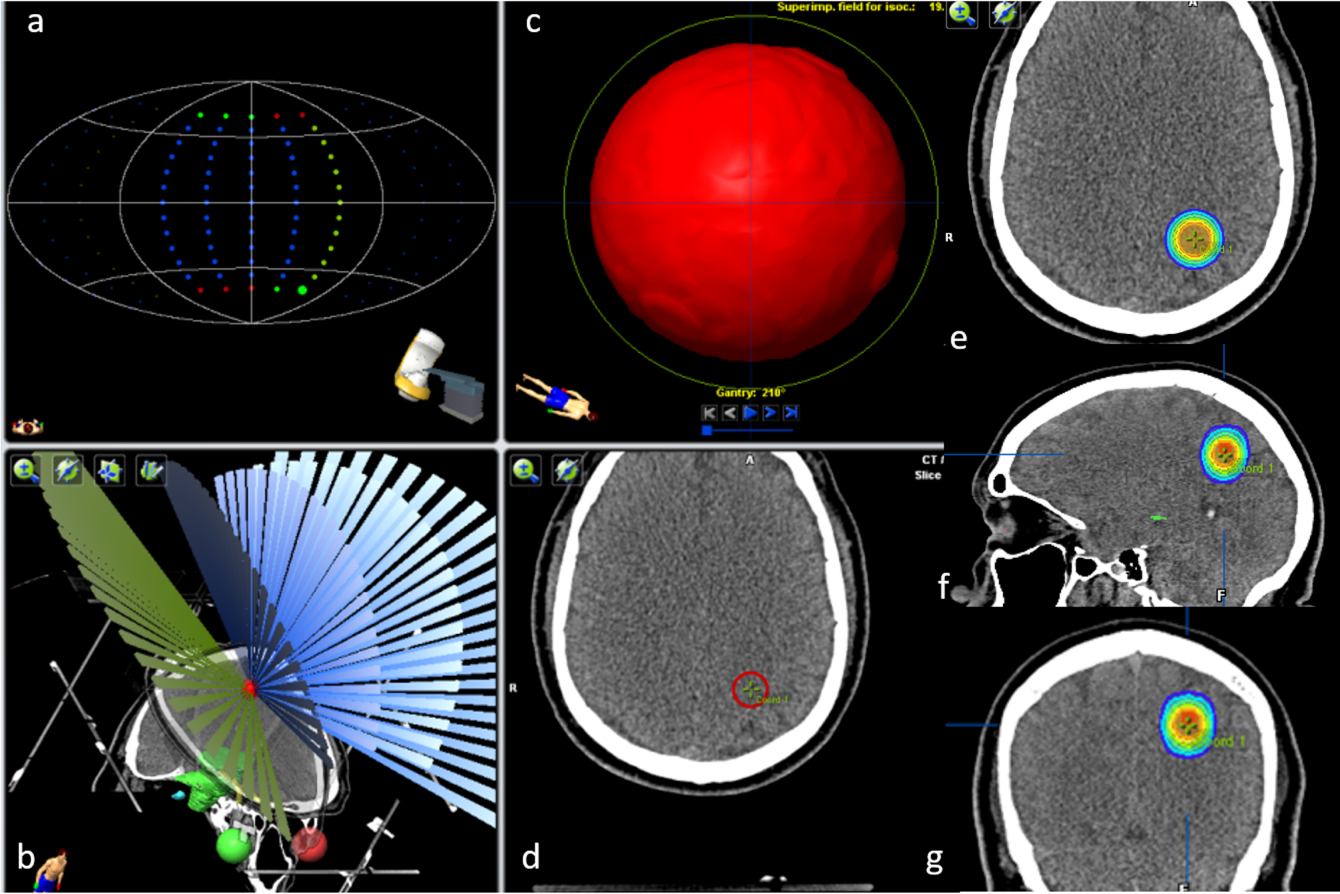
Simulated plan for a typical radiosurgery case using a treatment planning system (iPlan Dose v.4.1.1). a, b) Distributions of five non-coplanar arcs. c, d) Delineation of spherical lesions (in this case, the lesion volume is 0.107 cm^3^ for use with a 10.0-mm diameter circular collimator). e, f, g) Dose distribution (isodose lines) in three planes: axial, sagittal, and coronal, respectively.

The dose distribution calculation was performed in the iPlan RT TPS (v. 4.1.1, BrainLAB, Germany). The TPS used a Clarkson-type algorithm (Physics Manual, BrainLAB, Germany) to calculate dose distributions in patients when using circular collimators. Two beam profiles were created in the TPS using the corrected and uncorrected commissioned data. Dose distributions were calculated with these beam profiles in a simulated clinical treatment plan. The plan consisted of a set of computed tomography (CT) images (CT Hi-Speed scanner, GE Healthcare, USA) with a voxel size of 0.7 mm. Three spherical lesions were outlined with volumes of 0.004, 0.107, and 2.502 cm^3^, corresponding to the three circular collimated beams with 4.0, 10.0, and 20.0 mm to use the smallest, intermediate, and largest beam situation, respectively (Fig 2C and 2D).

Five non-coplanar arcs were used in the treatment plan for a total dose to the isocenter of 22.22 Gy with a prescription dose of 20 Gy (90% isodose line). The amplitude of each arc was 120° (see Fig 2A and 2B). The calculation grid size was set to 2 mm (see Fig 2E–2G). Note that García-Garduño et al. [2] previously showed that the gamma index criteria were not sensitive to the calculation grid size for circular cone-based radiosurgery.

### D. Analysis and comparison

#### D.I. Comparison and analysis of data set measurements and Monte Carlo simulation: TMR, OAR, and TSF

The TMR and TSF were compared in a point-by-point analysis of the relative differences. The corrected and uncorrected OAR were compared by applying a one-dimensional gamma analysis test to the measured profiles according to Technical Report Series (TRS) 430 [17]. The acceptance criteria used in this work were 1 mm/2%, 2 mm/10%, and 2 mm/30% for the inner, penumbra, and outer regions of the profiles, respectively. In addition, the FWHM of the field size, the 80%–20% penumbrae, and the 90%–10% beam penumbrae were measured. These measured data sets are referred to in this work as the uncorrected measurements.

#### D.II. Comparison and analysis of calculated dose distributions

Comparison of corrected and uncorrected dose distributions was performed using the gamma index test (DoseLab v. 4.11). The following gamma index criteria were used for the analysis: 1%/1 mm, 1%/3 mm, 1%/5 mm, 2%/2 mm, 2%/3 mm, and 3%/3 mm. The selection for the gamma index criteria was based on those reported in the literature [18–22]. Transversal profiles were acquired for the dose distributions. The profiles were exported for comparison with particular interest in the following dose regions: 100% to 80%, 80% to 20%, and <20% isodose lines for flat, gradient, and outer regions, respectively [22].

#### D.III. Comparison and analysis of dose volume histograms and monitor units

A set of dose volume histograms (DVH) of the lesions and the normal tissue was calculated for each simulated lesion using corrected and uncorrected data. The DVH were compared by calculating the percentage of the lesion volume covered by the dose prescription and the volume of normal tissue that received a dose of 12 Gy or higher (V12) according to the Quantitative Analyses of Normal Tissue Effects in the Clinic (QUANTEC) metric for evaluating normal tissue complications [23].

Finally, the percentage differences between the monitor units (MU) obtained with the corrected and uncorrected TSF were analyzed.

### E. Uncertainty budget

In this work, the main quantities of interest are the specific-detector correction factors as a function of depth and off-axis position as well as the calculated dose for the corrected and uncorrected situations. In the case of specific-detector correction factors, the adopted methodology for uncertainty estimations was based on that reported by Francescon et al. [24]. The main sources of uncertainty for Monte Carlo calculated quantities were statistical (dependent on the number of simulation histories), cross-section uncertainties, and the design and composition of the modeled detectors and radiation source in the simulation. For the TPS calculated dose, there were two situations of interest. First, for uncorrected dosimetry data, the source of uncertainty was the uncertainty associated with experimental measurements (OAR, TMR, and TSF) and their expansion in the TPS by the dose calculation algorithm. Second, for the corrected dosimetry data, the source of uncertainty was the same as that for the uncorrected data in addition to the uncertainty associated with the correction factors.

Table 1 shows the identified source uncertainty of the Monte Carlo calculated correction factors and an estimation of their magnitudes.

**Table 1:** Source uncertainty of the Monte Carlo calculated correction factors and estimation of their magnitudes.

Source of Uncertainty	Relative Magnitude	
	Type A	Type B
Statistical	Central: <0.8%*	
	Penumbra: <1.7%*	
	Outer: <16.0%*	
Cross-section		<0.40%**
Sensitive volume		<0.14%**
Wall thickness		<0.30%**
Wall density		<0.35%**
Total	Central: <1.02%	
	Penumbra: <1.81%	
	Outer: <16.00%	

*The magnitude was taken from the results of the Monte Carlo simulations for the absorbed dose expanded properly.

** Data were obtained from Med. Phys. 2011;38(12):6513–6527.

Table 2 shows the experimental sources of uncertainty for the dosimetry measurements and an estimation of their average magnitude for TMR, OAR, and TSF. The magnitude of the uncertainty associated with the TMR and OAR data was estimated by taking the average and standard deviation of five independent scans.

**Table 2:** Source uncertainty for the dosimetry measurements and estimation of their average magnitude for TMR, OAR, and TSF.

Source of Uncertainty	Relative magnitude		
	TMR	OAR	TSF
Reproducibility (includes type A and B uncertainties)	<0.30%	Central: <0.25%	<0.5%
		Penumbra: <4.0%	
		Outer: <10.0%	
LINAC output (type B)	-	-	<0.7%
Total	<0.30%	Central: <0.25%	<0.9%
		Penumbra: <4.0%	
		Outer: <10.0%	

From the data shown in Tables 1 and 2, the total uncertainty for the corrected and uncorrected data could be estimated as the expansion of the associated uncertainties of the experimental and Monte Carlo simulations.

Table 3 presents the estimated uncertainty of the calculated dose with and without the application of correction factors. The estimation was performed by assuming that the calculated dose had a spherical-like distribution. Then, the uncertainty was estimated for the central, penumbra, and outer regions of the distribution.

**Table 3:** Uncertainty in calculated dose for uncorrected and corrected data sets.

	Estimated Uncertainty in Calculated Dose*		
	Central (axis)	Penumbra	Outer
Uncorrected	<0.39%	<4.01%	<10.00%
Corrected	<1.09%	<4.40%	<18.87%

*The TPS uses the direct product of TPR, OAR and TSF to calculate the dose distribution in the patient according with BrainLAB Physics Reference Guide. The uncertainty was expanded accordingly.

## Results

### A. Determination of TMR, OAR and TSF: Measurements, Monte Carlo simulation and validation modeling

**Fig 3:**
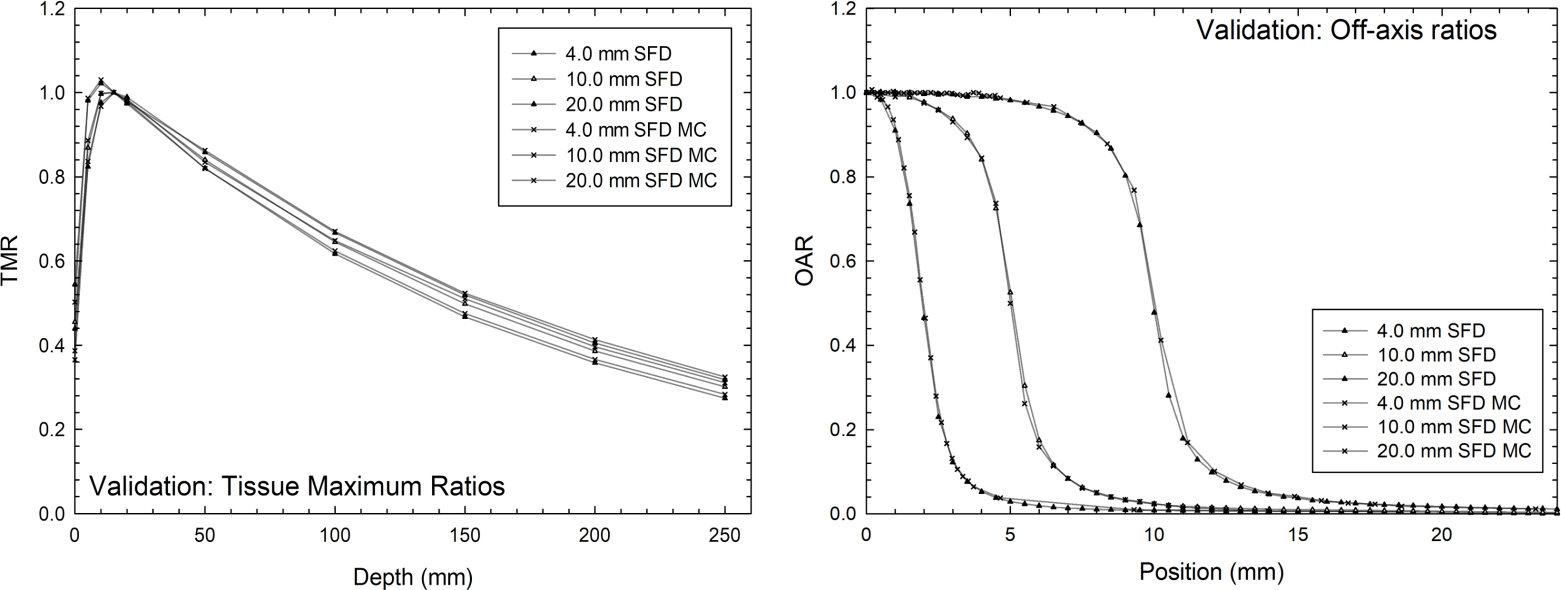
Comparison of TMR and OAR between Monte Carlo (MC) simulation of SFD and experimental measurements.

Fig 3 and Table 4 show the validation of the SFD Monte Carlo simulation comparison using the TMR, OAR, and TSF experimental data sets. These measurements and calculations are in good agreement with those reported in the literature [2,10,25,26].

**Table 4:** Validation of TSF between Monte Carlo (MC) simulation of SFD and experimental measurements. The reference value used to calculate de differences was the Monte Carlo calculated TSF.

	Total Scatter Factors			
Cone diameter (mm)	SFD	SFD MC	Difference (%)
4.0	0.664	0.662	0.30
10.0	0.885	0.874	1.24
20.0	0.953	0.951	0.21

The average differences for TMR between the SFD measures and Monte-Carlo-calculated SFD were 0.55%, 0.68%, and 0.63% for circular collimators of 4.0, 10.0, and 20.0 mm, respectively. For the OAR, the average differences were 0.57%, 0.48%, and 0.92% for 4.0, 10.0, and 20.0 mm, respectively. Finally, for TSF, a relative difference of ≤ 1.24% was obtained in all cases (see Table 4).

### B. Determination of detector-specific beam correction factors $k_{Q_{clin},\;Q_{msr}}^{f_{clin},\;f_{msr}}$

**Fig 4:**
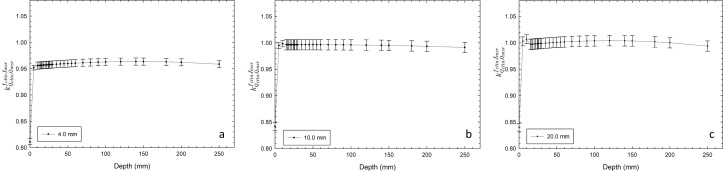
Correction factors $k_{Q_{clin},\;Q_{msr}}^{f_{clin},\;f_{msr}}$ to TMR for circular collimators of a) 4.0 mm, b) 10.0 mm, and c) 20.0 mm at the isocenter. Raw data is available in S1 File.

Fig 4 shows the variation of correction factors $k_{Q_{clin},\;Q_{msr}}^{f_{clin},\;f_{msr}}$ as a function of depth in water (TMR setup) for circular collimators of 4.0, 10.0, and 20.0 mm at the isocenter. The variation of all correction factors was less than 1% and was close to unity for the circular collimators of 10.0- and 20.0-mm. For the 4-mm circular collimator, the value of the correction factor was close to 0.950.

**Fig 5:**
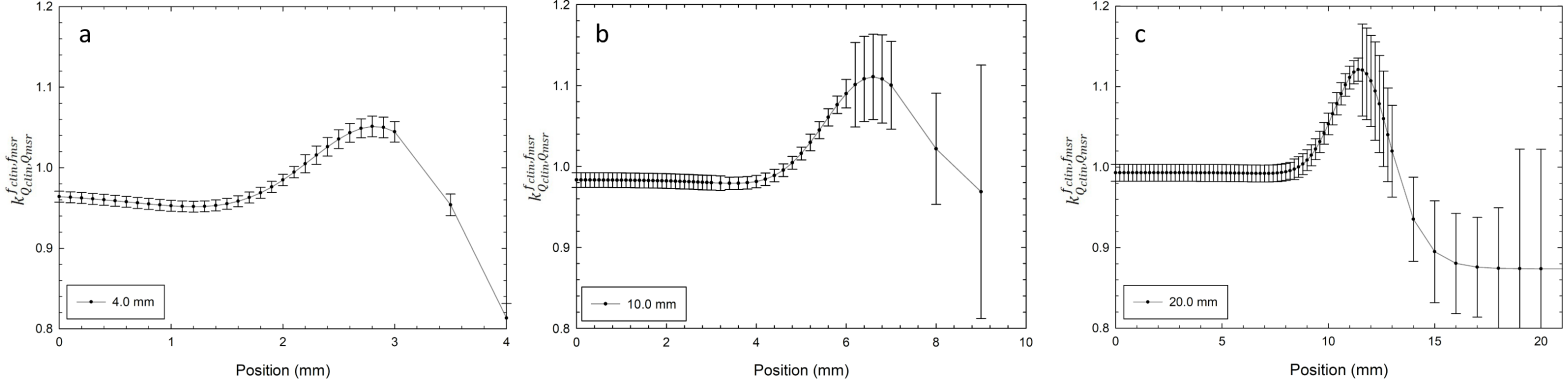
Correction factors $k_{Q_{clin},\;Q_{msr}}^{f_{clin},\;f_{msr}}$ to OAR for circular collimators of a) 4.0 mm, b) 10.0 mm, and c) 20.0 mm at the isocenter. Raw data is available in S1 File.

Fig 5 shows the variation of correction factors $k_{Q_{clin},\;Q_{msr}}^{f_{clin},\;f_{msr}}$ to OAR for circular collimators of 4.0, 10.0, and 20.0 mm at the isocenter.

The behavior of these specific correction factors for TMR and OAR were similar to those reported by Francescon et al. [10]. This behavior for the circular collimators of 4.0 and 20.0 mm used in this work was remarkably similar to those reported by Francescon et al. [10] for circular collimators of 5.0 and 25 mm, respectively.

Finally, the correction factors $k_{Q_{clin},\;Q_{msr}}^{f_{clin},\;f_{msr}}$ of TSF and comparison to results reported in the literature are shown in Table 5.

**Table 5:** TSF measurements with SFD and $\;k_{Q_{clin},\;Q_{msr}}^{f_{clin},\;f_{msr}}$ calculated with Monte Carlo simulation.

Accelerator Type	Field size (mm)	Measurements with SFD diode	$k_{Q_{clin},\;Q_{msr}}^{f_{clin},\;f_{msr}}$	Percentage Differences TSF (%)	Percentage Differences $k_{Q_{clin},\;Q_{msr}}^{f_{clin},\;f_{msr}}$
This work	4	0.664	0.982		
Novalis		0.651	0.975	1.96	0.71
This work		0.885	0.997		
Novalis*	10	0.882	1.005	0.34 (Novalis)	0.80
CyberKnife*		0.867	1.005	2.03(Cyberknife)	-
This work	20	0.953	1.004		
CyberKnife*		0.950	1.005	0.31	0.10

*Comparative data were taken from Med. Phys 40(7): 071725-1-13 (2013).

The percentage difference between the specific correction in this work and those of Bassinet [25] were 0.71%, 0.80%, and 0.10% (all below to 1%) for circular collimators of 4.0, 10.0, and 20.0 mm, respectively.

### C. Analysis and comparison

#### C.I. Comparison and analysis of data set measurements and Monte Carlo simulation: TMR, OAR, and TSF

**Fig 6:**
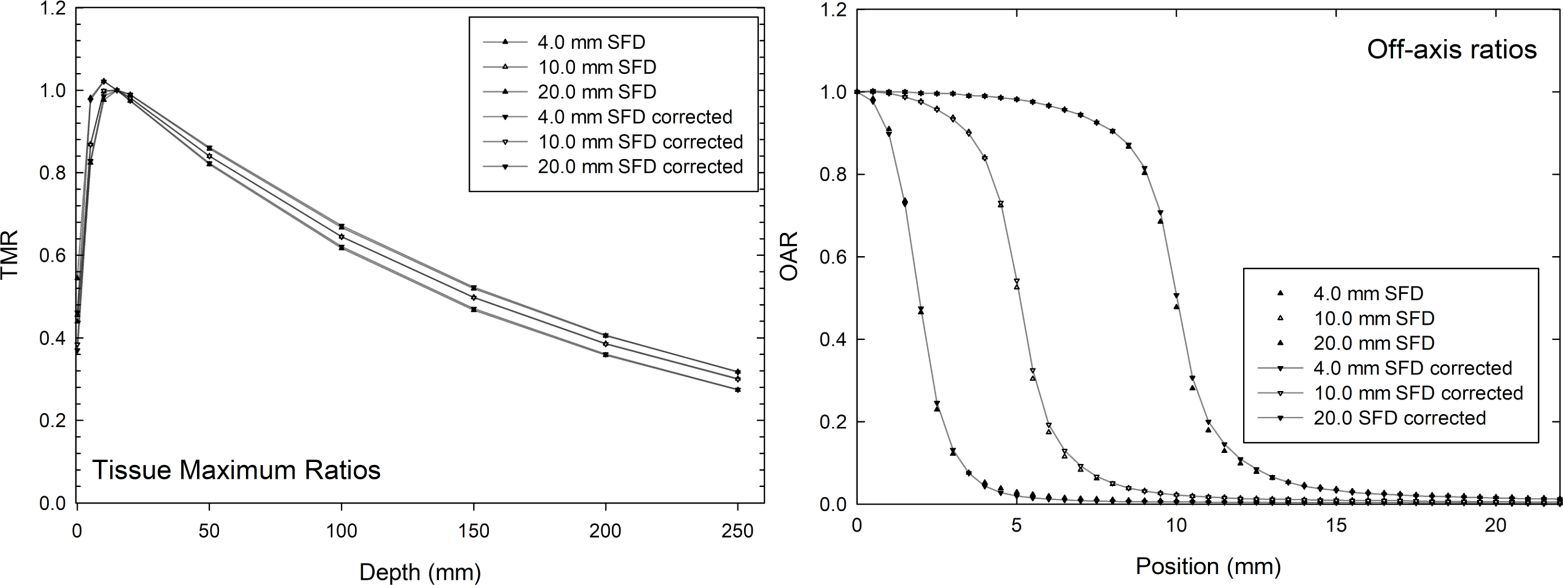
Comparison of TMR and OAR for uncorrected and corrected SFD measurements.

Fig 6 shows a comparison between uncorrected and corrected data measured by the SFD detector. The average differences between TMR data sets directly measured and corrected are 1.78%, 2.68%, and 2.59% for circular collimators of 4.0, 10.0, and 20.0 mm, respectively. The comparison with OAR is shown in Table 6. The 80–20% and 90–10% penumbrae show the higher differences between corrected and uncorrected data. On the other hand, the FWHM values are very similar in magnitude for the corrected and uncorrected profiles.

**Table 6:** Results of FWHM, the 80%–20% penumbrae and 90%–10% penumbrae to OARs between uncorrected and corrected SFD measurements.

Cone (mm)	4.0		10.0		20.0	
OARs	SFD	SFD Corrected	SFD	SFD Corrected	SFD	SFD Corrected
Penumbrae 80–20%	1.265	1.352	1.658	1.746	1.849	1.908
Penumbrae 90–10%	2.167	2.260	3.176	3.397	3.892	4.081
FHWM (mm)	3.866	3.899	10.116	10.197	19.895	20.036

Table 7 shows the results of the one-dimensional gamma-analysis performed on the OARs. The major differences are observed in the outer region where the percentage of points passing the gamma index test decreases to 79.487–83.674%.

**Table 7:** Gamma index analysis test of the measured profiles according to TRS 430 [17] using the uncorrected SFD measurement as reference. Data show the percentage of the points with γ(Δδ, ΔD) ≤ 1.

Cone (mm)	4.0	10.0	20.0
Region	SFD-corrected	SFD-corrected	SFD-corrected
Inner	100.0	100.0	100.0
Penumbrae	100.0	100.0	100.0
Outer	80.0	83.674	79.487

As a shown in Table 7, the results for the outer region do not pass the gamma test for any circular collimator, where a threshold of 90–95% is considered acceptance. Finally, Table 8 shows a comparison between the corrected and uncorrected TSF measurements, as well the percentage difference.

**Table 8:** TSF and the percentage difference between uncorrected and corrected SFD measurements. The reference value used to calculate the differences was the corrected TSF.

	Total Scatter Factors		Percentage difference (%)
Cone diameter (mm)	SFD	SFD corrected	SFD-corrected
4.0	0.664	0.652	1.84
10.0	0.885	0.883	0.23
20.0	0.953	0.957	0.42

The highest difference between the TSF measurements occurs for the smaller circular collimator (4.0 mm), which shows a 1.840% percentage difference. The larger circular collimators (10.0 and 20.0 mm) show percentage differences of less than 0.42%.

#### C.II. Comparison and analysis of calculated dose distributions (planning simulation)

**Fig 7:**
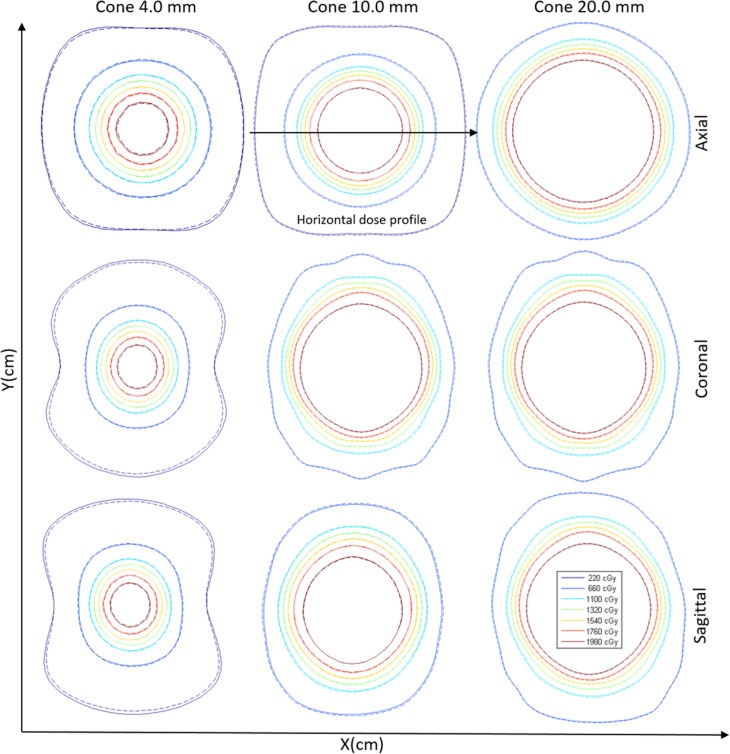
Comparison dose distributions between corrected and uncorrected dosimetry data sets. Solid lines are the dose distributions obtained with uncorrected data. Dashed contour lines are dose distributions obtained with data corrected with detector-specific beam correction factors.

A qualitative comparison of the gamma index analysis between the dose distribution obtained with corrected and uncorrected SFD measurements is presented in Fig 7.

The results of the gamma index analysis are shown in Table 9 for the following criteria: 1%/1mm, 1%/3mm, 1%/5mm, 2%/2mm, 2%/3mm, and 3%/3 mm.

**Table 9:** Gamma index results for the criteria of 1%/1mm, 1%/3mm, 1%/5mm, 2%/2mm, 2%/3mm, and 3%/3 mm comparing dose distributions between corrected and uncorrected SFD measurements.

	Cone 4.0 mm					
Plane	1%/1mm	1%/3mm	1%/5mm	2%/2mm	2%/3mm	3%/3mm
Axial	100	100	100	100	100	100
Coronal	100	100	100	100	100	100
Sagittal	100	100	100	100	100	100
	Cone 10.0 mm					
Plane	1%/1mm	1%/3mm	1%/5mm	2%/2mm	2%/3mm	2%/3mm
Axial	84.3	100	100	100	100	100
Coronal	95.6	100	100	100	100	100
Sagittal	98.9	100	100	100	100	100
	Cone 20.0 mm					
Plane	1%/1mm	1%/3mm	1%/5mm	2%/2mm	2%/3mm	2%/3mm
Axial	82	100	100	100	100	100
Coronal	85.3	100	100	100	100	100
Sagittal	88	100	100	100	100	100

**Fig 8:**
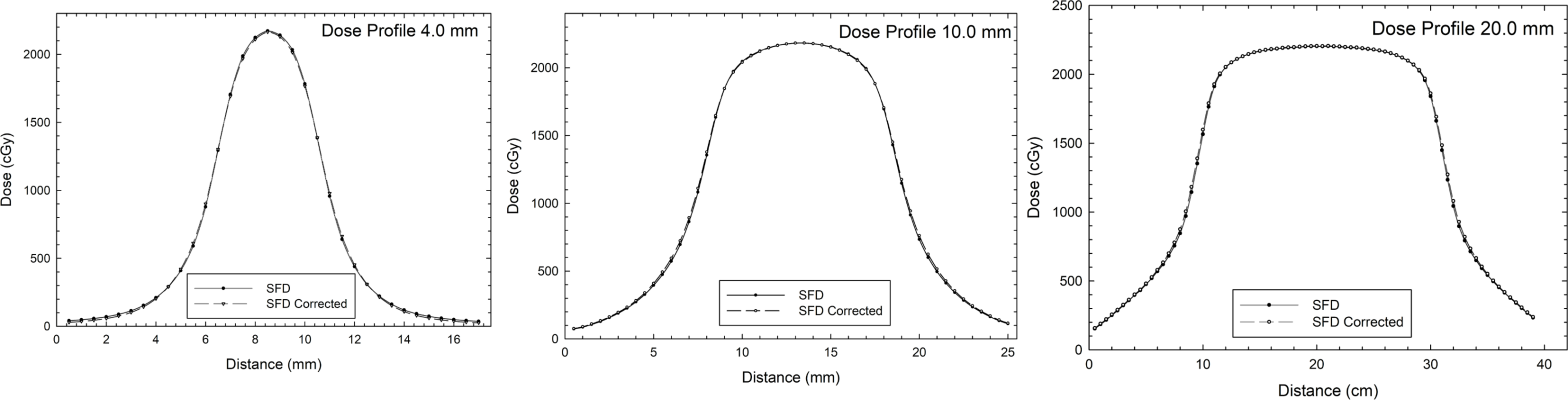
Horizontal dose profile calculated with a 0.5-mm grid calculation matrix for two data sets: Uncorrected and SFD data corrected by detector-specific beam correction factor $\left(k_{Q_{clin},Q_{msr}}^{f_{clin},f_{msr}}\right)$.

The results of the gamma index comparison show that 100% of the points for all circular collimators satisfy all gamma criteria, except for the 1%/1 mm criteria for the circular collimators of 10.0- and 20.0-mm. Finally, the results of three central dose profiles (Fig 7) were exported to analyze the behavior for the flat, gradient, and outer profile regions. These results are shown in Fig 8.

In Fig 8, the behavior of profiles is practically the same for both data sets in all cases and regions (flat, gradient, and outer).

#### C.III. Comparison and analysis of dose volume histograms and monitor units

Table 10 presents the percentage of the lesion volume covered by the dose prescription and the volume of normal tissue that received a dose of 12 Gy or higher for the uncorrected SFD data set and the SFD data set corrected by detector-specific beam correction factors $\left(k_{Q_{clin},Q_{msr}}^{f_{clin},f_{msr}}\right)$.

**Table 10:** Percentage of the lesion volume covered by the dose prescription and the volume of normal tissue that received a dose of 12 Gy or higher for corrected and uncorrected SFD measurements.

	DVH Target volume (%) (Dose Prescription)			DVH Normal Tissue (%) (≥12 Gy)		
Cone size (mm)	SFD	SFD Corrected	Differences	SFD	SFD Corrected	Differences
4.0	100	96.875	3.125	0.003	0.003	0.000
10.0	100	100	0.000	0.017	0.019	0.002
20.0	100	100	0.000	0.103	0.105	0.002

For the larger circular collimators (10.0 and 20.0 mm), there is no difference in the coverage of the target volume, and the difference found in normal tissue is 0.002% in both cases. On the other hand, in the case of the smallest circular collimator (4.0 mm), the difference in coverage is 3.125%. Table 11 shows the MU per arc and the percentage difference between the uncorrected and corrected SFD measurements.

**Table 11:** MU per arc and percentage difference of two data sets: Uncorrected and corrected SFD measurements.

	Monitor Units						Percentage difference (%)		
	Cone 4.0 mm		Cone 10.0 mm		Cone 20.0 mm		Cone (mm)		
Arc	SFD	SFD Corrected	SFD	SFD Corrected	SFD	SFD Corrected	4.0	10.0	20.0
1	1080	1180	596	645	587	629	8.5	7.6	6.7
2	918	1004	620	672	546	585	8.6	7.7	6.7
3	784	860	664	719	517	554	8.8	7.6	6.7
4	716	786	718	778	504	541	8.9	7.7	6.8
5	687	755	751	814	505	542	9.0	7.7	6.8

These results show the largest percentage differences in this work. The percentage differences ranged from 6.7 to 9.0% and increase as the field size decreases. The greatest difference of deposited monitor units (9.0%) occurs with a 4.0-mm cone.

## Discussion

In this work, we evaluated the impact of detector-specific beam correction factors on the calculated dose distribution in static non-conventional photon beams using circular collimators. To this end, the study was divided into three parts. In the first part, dosimetric data were measured using an SFD for three circular collimators. In the second part, a set of detector-specific correction factors were calculated using Monte Carlo simulations following the methodology proposed by Alfonso et al. [9] and Francescon et al. [10] for the same set of circular collimators. Finally, the third part incorporated the commissioned data sets into the TPS in both corrected and uncorrected form. The TPS was used to calculate the dose distributions in a clinical situation. The resulting dose distributions (corrected and uncorrected) were compared and analyzed through the gamma index, DVH metrics, and MU.

The SFD measurements from dosimetric data and Monte Carlo simulation (TMR, OAR, and TSF) showed good agreement with the literature [2,25,26]. In particular, the TSFs reported in this work were very similar to those reported by Bassinet et al. [25] and measured with SFD on a Novalis LINAC (for circular collimators of 4.0 and 10.0 mm) and a CyberKnife (for circular collimators of 10.0 and 20.0 mm). The percentage difference between the TSF values in this work and those of Bassinet was less 2.03% [25].

The calculated correction factors $k_{Q_{clin},\;Q_{msr}}^{f_{clin},\;f_{msr}}$ for the central axis were compared with those reported by other authors with similar detector and linac setups [25]. The correction factors $k_{Q_{clin},\;Q_{msr}}^{f_{clin},\;f_{msr}}$ showed a maximum percentage difference of 0.80% when applied to TSF. These results also supported the statement that the detector-specific correction factors have low sensitivity to small changes in beam quality. The behavior of these specific correction factors to TMR and OAR were similar to those reported by Francescon et al. [10]. In particular, the behavior for the circular collimators of 4.0 and 20.0 mm of this work was remarkably similar to that reported by Francescon et al. for circular collimators of 5.0 and 25 mm, respectively. However, a quantitative comparison of these specific correction factors was not possible because different field sizes were used in calculating these factors.

The analysis and comparison between the data set measured directly with the SFD and the data set corrected by the detector-specific beam correction factors $\left(k_{Q_{clin},Q_{msr}}^{f_{clin},f_{msr}}\right)$ showed excellent agreement for all considered circular collimators. The largest percentage difference for TMR results (2.59%) was found for the 20.0-mm circular collimator. However, this difference was lower than the impact of varying the detector type used for the dosimetry of non-conventional fields (greater than 6.21%) [2].

Good agreements between the two data sets were also obtained among the OAR results for all circular collimators. This observation was supported by the analysis of the penumbrae, FWHM, and the gamma index test. All penumbrae (80–20% and 90–10%) and the FWHM were very similar in magnitude for both data sets. The data sets corrected by the detector-specific correction factors were systematically greater than the data set without correction. For both data sets in the inner and penumbrae regions, 100% of the points passed the gamma test. In contrast, none of the OAR passed the acceptance criteria of 90–95% for the outer region. Thus, in the outer region, the profiles were not very similar owing to the magnitude of increase of detector-specific corrections factors in the outer region compared with that of the penumbrae and inner regions.

The TSF values were compared between both sets. The greatest difference between the TSF values (1.84%) occurred for the smaller circular collimator (4.0 mm). For the larger circular collimators (10.0 and 20.0 mm), the percentage differences were less than 0.42%. However, while these differences were relatively small, the monitor units that determine the delivered dose to the patient must be calculated using the TSF. Therefore, evaluating the differences in the monitor units provided by the TSF in each case (without and with detector-specific beam correction factors) was necessary because these differences may not have been reflected with the same proportionality when the monitor units were calculated.

For the gamma test analysis, only the extreme case of 1%/1 mm was used. It showed that the profiles for the circular collimators of 10.0- and 20.0-mm failed the test. However, this scenario did not represent possible clinical situations since 2 mm/3%, 2 mm/2%, 1mm/5%, and 1 mm/3% are more commonly suggested in clinical practice [20]. In the calculation of the dose distributions, the TSF was directly related to the deposited dose through the MU calculation, while the TMR and OAR were involved in the behavior of the dose distributions in any treatment planning. Therefore, from the results of the gamma index criteria, the use of detector-specific beam correction factors $\left(k_{Q_{clin},Q_{msr}}^{f_{clin},f_{msr}}\right)$ for TMR and OAR did not impact the calculated dose distribution for SFD. This is because the evidence provided by this work suggested that the calculations performed by the TPS had a low sensitivity to these differences.

The DVH comparison showed that, for the 4.0-mm collimator case, the V12 difference between the corrected and uncorrected dose distributions was 3.125%. For normal tissue, a difference of ≤0.002% was found for the circular collimators of 10.0- and 20.0-mm. The importance of the difference found in the dose coverage of the smallest circular collimator may have depended on the prescribed dose considered for the treatment, which normally is between 95 and 98%.

Finally, the monitor units per arc and the percentage difference between the uncorrected and corrected measurements were determined. These results showed the largest percentage differences. Moreover, these differences increased up to 9.0% as the field size decreased and may lead to important differences between calculated and delivered dose to the patient. The International Commission on Radiation Units and Measurements (ICRU) recommends that the overall accuracy in the radiation dose delivered to the patient is within 5% [17]. On the other hand, the choice of a tolerance value could be dependent on the uncertainty attributed to the reference data, which could be dependent on the clinical situation. However, the tolerance levels associated with small-field treatments will be substantially more restrictive than those for conventional treatments [17]. For that reason, wide differences in the monitor units suggested that the detector-specific beam correction factors $\left(k_{Q_{clin},Q_{msr}}^{f_{clin},f_{msr}}\right)$ should be used. These results were reinforced by the fact that the use of the formalism proposed by Alfonso et al. [9] for unconventional fields in the case of the TSF for this treatment planning system was necessary.

To generalize the results presented in this study, a comprehensive equivalent work is necessary with other TPSs, calculation algorithms, and detector types. Dose calculation algorithms in modern radiation therapy mainly use relative measurements (TSF, TMR/PDD, OAR) to determine the initial parameters for radiation transport and verify the accuracy of the algorithm to calculate the dose in water. These algorithms model the transport of ionizing radiation through matter by convolution techniques, direct solution of the Boltzmann equation or Monte Carlo simulations. On the contrary, empirical based dose calculation algorithms (as used in this work) directly depend on the experimental measurements.

Furthermore, the commissioning of these algorithms often involves “fine tuning” of the transport parameters to match calculation with experimental measurements. Moreover, these algorithms generally only require relative measurements for field sizes down to 3 cm × 3 cm potentially compromising the accuracy of the dose calculation for small fields. Owing to the complexity of modern TPSs, it is not straightforward to observe the possible impact of the application of the correction factors on the calculated dose. It is necessary to perform a comprehensive study to understand, for example, the effects of the correction factors in 1) spectral and electron contamination determination, 2) focal spot size (particularly in small fields), 3) monitor chamber backscatter factors and 4) the impacts of the three preceding factors in the calculated dose.

## Conclusions

This work presented three important conclusions for the use of detector-specific beam correction factors $\left(k_{Q_{clin},Q_{msr}}^{f_{clin},f_{msr}}\right)$ in a treatment planning system, specifically for an SFD and iPlan RT with the Clarkson algorithm (v. 4.1.1, BraniLAB, Germany) for circular collimated beams:

1) Detector-specific correction factors shown low sensitivity to small changes in beam quality.
2) The use of detector-specific beam correction factors $\left(k_{Q_{clin},Q_{msr}}^{f_{clin},f_{msr}}\right)$ for TMR and OAR does not have an important impact on the calculated dose distribution by the TPS. The latter is valid for the dose calculation algorithm and detector used in this work.
3) The use of detector-specific beam correction factors $\left(k_{Q_{clin},Q_{msr}}^{f_{clin},f_{msr}}\right)$ for TSF should follow the formalism proposed by Alfonso et al. [9] to ensure the MUs delivered to the patient are accurate.

Finally, to verify if these results may be generalized, a rigorous and comprehensive equivalent study is required for other TPSs, calculation algorithms, and detector types.

## Supporting information

S1 File Monte Carlo simulation and correction factors. Compressed file containing a description of the methodology used to perform the Monte Carlo simulations and raw data from Figs 4 and 5.
(ZIP)
