# Supplementary material for: Detector-specific correction factors in radiosurgery beams and their impact on dose distribution calculations
Source: PLoS One. 2018 May 15;13(5):e0196393. doi: 10.1371/journal.pone.0196393 (PMC5953445; doi:10.1371/journal.pone.0196393)
Supplement: S1 File — Compressed file containing a description of the methodology used to perform the Monte Carlo simulations and raw data from Figs 4 and 5. (ZIP) [file pone.0196393.s001.zip › Supporting Files/S1_Text.docx]

**S1_Text Simulation of the Novalis linear Accelerator to produce small fields with circular collimators**

The simulation was carried out using the EGSnrc and BEAMnrc Monte Carlos libraries in their version 2016. Due to confidentiality issues, it is no possible to publish the geometry of the LINAC. However, final phase space files may be provided upon request. The combined size of the 3 phase space files (one for each circular collimator) is about 800 MB.

The simulation of the LINAC was performed following the methodology used by Popescu et al. (2005) [1]. Essentially, the simulation (and the LINAC) is divided in two parts. The first part simulates all the fixed components of LINAC: target, primary collimator, flatting filter, ionization chamber and mirror. A big phase space is scored at the end of the LINAC mirror and prior to the collimator jaws. Popescu and co-workers call this space file as Phase Space A (PS_A_). The second part involves the simulation of the “moving” parts of the linac and accessories: jaws, multileaf collimator, circular cones, electron cones, wedges, etc. A second phase space file is scored at the end of the accessories and/or collimator and it is called Phase Space B (PS_B_). In our case, the phase space file B is scored at the end (the face near to the patient) of the circular collimator.

In our work, 3 phase space files were generated and used to calculate the dose in water and in the detector. The dose calculation was performed using the DOSRZnrc use code version 2016. The central axis calculations (tissue maximum ratios and the total scatter factors) are straightforward to calculate. For off-axis ratios, we use the SOURCE 23 in DOSRZnrc. The SOURCE 23 allows link the LINAC head simulation to the simulation of the detector. The SOURCE 23 has the advantage that it is possible to perform displacements of the source along the x and y axes. The later allows to move the source (LINAC head) and simulate the profile point by point. Each point for each dose profile is an independent simulation.

[1] Popescu IA, Shaw CP, Zavgorodni SF, Beckham WA. Absolute dose calculations for Monte Carlo simulations of radiotherapy beams. Phys Med Biol 2005;50:3375–92. doi:10.1088/0031-9155/50/14/013.
